# Maternal interventions to decrease stillbirths and neonatal mortality in Tanzania: evidence from the 2017-18 cross-sectional Tanzania verbal and social autopsy study

**DOI:** 10.1186/s12884-023-06099-y

**Published:** 2023-12-11

**Authors:** Henry D. Kalter, Alain K. Koffi, Jamie Perin, Mlemba A. Kamwe, Robert E. Black

**Affiliations:** 1 Department of International Health, Institute for International Programs, Johns Hopkins Bloomberg School of Public Health, Baltimore, MD United States of America; 2 Department of International Health, Health Systems, Johns Hopkins Bloomberg School of Public Health, Baltimore, MD United States of America; 3 National Bureau of Statistics, Dodoma, United Republic of Tanzania

**Keywords:** Stillbirth, Intrapartum stillbirth, Neonatal mortality, Maternal complications, Hospital delivery, C-section, Verbal and social autopsy

## Abstract

**Background:**

Reduction of Tanzania’s neonatal mortality rate has lagged behind that for all under-fives, and perinatal mortality has remained stagnant over the past two decades. We conducted a national verbal and social autopsy (VASA) study to estimate the causes and social determinants of stillbirths and neonatal deaths with the aim of identifying relevant health care and social interventions.

**Methods:**

A VASA interview was conducted of all stillbirths and neonatal deaths in the prior 5 years identified by the 2015-16 Tanzania Demographic and Health Survey. We evaluated associations of maternal complications with antepartum and intrapartum stillbirth and leading causes of neonatal death; conducted descriptive analyses of antenatal (ANC) and delivery care and mothers’ careseeking for complications; and developed logistic regression models to examine factors associated with delivery place and mode.

**Results:**

There were 204 stillbirths, with 185 able to be classified as antepartum (88 [47.5%]) or intrapartum (97 [52.5%]), and 228 neonatal deaths. Women with an intrapartum stillbirth were 6.5% (adjusted odds ratio (aOR) = 1.065, 95% confidence interval (CI) 1.002, 1.132) more likely to have a C-section for every additional hour before delivery after reaching the birth attendant. Antepartum hemorrhage (APH), maternal anemia, and premature rupture of membranes (PROM) were significantly positively associated with early neonatal mortality due to preterm delivery, intrapartum-related events and serious infection, respectively. While half to two-thirds of mothers made four or more ANC visits (ANC4+), a third or fewer received quality ANC (Q-ANC). Women with a complication were more likely to deliver at hospital only if they received Q-ANC (neonates: aOR = 4.5, 95% CI 1.6, 12.3) or ANC4+ (stillbirths: aOR = 11.8, 95% CI 3.6, 38.0). Nevertheless, urban residence was the strongest predictor of hospital delivery.

**Conclusions:**

While Q-ANC and ANC4 + boosted hospital delivery among women with a complication, attendance was low and the quality of care is critical. Quality improvement efforts in urban and rural areas should focus on early detection and management of APH, maternal anemia, PROM, and prolonged labor, and on newborn resuscitation.

**Supplementary Information:**

The online version contains supplementary material available at 10.1186/s12884-023-06099-y.

## Background

 Reducing neonatal mortality (NNM) remains the greatest challenge globally to achieving the United Nations child mortality Sustainable Development Goals by the target year 2030 [1]. The contribution of neonatal deaths to under-five mortality (U5M) increased from 40% to 1990 to 45% in 2019 due to relatively greater success in overcoming childhood infectious diseases than maternal and perinatal complications. Sub-Saharan Africa (SSA) in 2019 had the world’s highest U5M and NNM rates, 75 and 27 deaths/1,000 live births, accounting for 53% and 42% of global under-five and neonatal deaths, respectively [2]. Stillbirth also remains a severe problem in SSA, with the world’s highest rate, 22/1,000 births in 2019, accounting for 44% of global stillbirths [3]. Just over half (51%) of these deaths in SSA are estimated to occur intrapartum, the period from the onset of labor until delivery [4].

Pregnancy and labor and delivery (L/D) complications are the most important risk factors for perinatal mortality (PNM) [5–10], with care provided during L/D affording the greatest mortality reductions for neonates [11, 12] and prevention of stillbirths [12]. Intrapartum care is most effectively delivered in a health facility, with higher level facilities providing basic (BEmONC) and comprehensive emergency obstetric and newborn care (CEmONC) best positioned to contribute to maternal, perinatal and neonatal survival [11, 12]. Quality antenatal care (ANC) also plays an important role in reducing PNM and NNM, directly through provision of efficacious interventions [11–14], and indirectly by promoting institutional delivery and educating women on danger signs of pregnancy and where to go for a complication [15, 16].

In 2019 and 2018, respectively, Tanzania had the ninth and tenth highest numbers of stillbirths [3] and neonatal deaths [17] in the world. From 1990 to 2019 Tanzania reduced its U5M rate by more than two-thirds, from 165 to 50/1,000, but NNM decreased only by half, from 40 to 20/1,000 [2]. Tanzania has tracked NNM and PNM through periodic Demographic and Health Surveys (TDHS) since 2004. The PNM rate decreased from 42 to 36/1,000 in the 2004 to 2010 surveys [18, 19], but then increased to 39/1,000 in the 2015-16 survey [20]. The contribution of stillbirths to PNM remained stable over this period, respectively 44.4%, 47.8% and 46.6%. The TDHS does not distinguish antepartum from intrapartum stillbirth.

 While skilled attendance at birth and emergency obstetric care made the largest contribution (29%) to the reduction in NNM in Tanzania from 2000 to 2012 [21], both the stagnant stillbirth rate and insufficient decrease in NNM have been attributed to failures in accomplishing critical maternal and neonatal care objectives of the country’s 2008-15 National Road Map Strategic Plan to Accelerate Reduction of Maternal, Newborn and Child Deaths (One Plan). In particular, inequities in health facility and cesarean (C-section) delivery (as a proxy for CEmONC) in rural areas and by socioeconomic status (SES) prevented achievement of the objectives [21]. As documented by the country’s 2016–2020 One Plan II program, by 2015 the country had achieved just 25% instead of the targeted 70% BEmONC coverage at health centers and 73% instead of 100% CEmONC coverage at hospitals [22]. In addition, while 63% of deliveries took place in a health facility and 6% were by C-section, there was a 32% urban/rural gap and 53% SES gap in facility deliveries, and 8% urban/rural and 14% SES gaps in C-sections [20].

We conducted a national verbal and social autopsy (VASA) study of stillbirths and under-five deaths to estimate the causes and social determinants of the deaths to provide evidence for the country to consider in developing its maternal, newborn and child health programming. We previously reported on the neonatal and 1-59-month-old causes of death (COD) and preventive and curative indicators [23]. The current analysis aims to differentiate antepartum and intrapartum stillbirth and assess the contribution of provider delay in conducting C-section to intrapartum stillbirth; identify maternal complications associated with antepartum and intrapartum stillbirth and leading causes of neonatal death; and examine the impact of different aspects of ANC and its interaction with complications on hospital delivery; all to provide evidence needed to focus antenatal and intrapartum interventions targeted at decreasing stillbirths and neonatal deaths.

## Methods

The VASA study was conducted on the platform of the 2015-16 TDHS of 13,360 households [20]. The TDHS included a lifetime birth history of all married women 15-49-years-old to identify all live births and deaths, as well as specific questions on ‘pregnancy terminations’ that did not end in a live birth.

The VASA study was conducted from mid-November through December 2017, with a follow-up round from January-February 2018 to locate respondents who had moved from their original location. Integrated VASA interviews were attempted of all 851 7-plus-months pregnancy terminations and neonatal (0–27 days) and 1-59-month-old deaths in the five years prior to their TDHS interview. Our prior publication provides details on the VASA questionnaire and study implementation [23].

### Birth status, cause of neonatal death and maternal complications

The VASA interview first evaluated possible TDHS misclassification of 7-plus-months pregnancy terminations (TDHS stillbirths) and deaths of live born children by asking about cardinal signs of life at birth not asked about by the TDHS. A child was considered stillborn if reported to have never cried, moved, or breathed. Live-born children were classified as a neonatal or 1-59-month-old death, depending on the VASA-reported age at death. Our previous VASA analysis [23] directly estimated the neonatal COD discussed in the current paper using the expert algorithm method [23–25]. Prior estimates of neonatal COD in Tanzania have utilized a multinomial logistic regression model with global VA data and national proximate covariates as inputs [26].

An intrapartum stillbirth was defined as one in which the mother reported that the baby either did not stop moving before labor began, or last moved less than one hour before labor began or less than eight hours before delivery. We also examined how using a 12-hour cut-off and/or including report of no maceration (as an ‘or’ statement) might alter the antepartum and intrapartum proportions and their apparent misclassification. Because the utilized VA questionnaire does not include a question on the time before delivery the mother last felt the baby move, this was determined (for women whose babies stopped moving before labor began) by summing the time before labor began that the baby last moved plus labor duration. The intrapartum category also excludes stillbirths with severe congenital abnormalities since it is surmised that such deaths were not due to complications of the birth process.

We defined pregnancy (before labor onset) and L/D complications using algorithms of illness signs and symptoms (panel). For the logistic regression analyses of delivery place and mode described below, “any complication” was defined, respectively, as having one or more pregnancy complications or one or more L/D complications that started before reaching a delivery facility, and as having one or more pregnancy or L/D complications.

### Panel definitions of maternal complications

**Table:** 

**Pregnancy complications (start before labor onset)**
*Antepartum hemorrhage*: Any vaginal bleeding before the onset of labor
*Preeclampsia/eclampsia*: (Puffy face and (blurred vision or severe headache or high blood pressure)) and/or (Convulsions and no fever and no history of convulsions)
*Maternal infection* *:* Fever and (severe abdominal pain or smelly vaginal discharge)
*Maternal anemia*: (Severe anemia or (pallor and shortness of breath)) and (too weak to get out of bed or fast or difficult breathing)
*Maternal diabetes*: Diabetes that started before or during the pregnan*cy*
*Premature rupture of membranes*: Water broke 6 hours or more before labor began
*Malaria*: Convulsions and fever
**Labor/delivery complications (start after labor onset)**
*Intrapartum hemorrhage*: Excessive bleeding during labor or delivery
*Preeclampsia/eclampsia: same as for pregnancy*
*Maternal infection*: Fever and (severe abdominal pain or smelly vaginal discharge or foul smelling amniotic fluid)
*Maternal anemia*: same as for pregnancy
*Prolonged labor*: Labor for 12 or more hours
*Malpresentation*: Child delivered not head first
*Cord complication*: Cord delivered first or cord around the child’s neck

### Statistical analyses

The CSPro data collected on netbooks were converted to SAS 9.4 [27] and STATA 16.0 [28] datasets for analysis. Following determination of stillbirths and neonatal deaths, all subsequent analyses were conducted of data weighted and design-corrected based on the TDHS multi-stage sampling design. The same was true for the neonatal COD identified by the earlier paper [23], now utilized to examine maternal complications-neonatal COD associations. For simplicity of presentation, the weighted and survey design-corrected fractional frequencies were rounded up to the next higher level. Descriptive statistics included frequency distributions. Tests of association included odds ratios (OR) and adjusted odds ratios (aOR) with 95% confidence interval, and Pearson or Rao-Scott chi-square.

We examined the association of pregnancy and L/D complications with known causes of antepartum [29–36] and intrapartum [31–33, 37, 38] stillbirth and separately for three main causes of neonatal death, including preterm delivery [32, 39–43], intrapartum-related events (birth asphyxia, birth injury) [38, 41, 44–48] and serious neonatal infection [39, 40]. These were also the leading causes in our study population [23]. We conducted descriptive analyses of ANC coverage and mothers’ careseeking for pregnancy and L/D complications, as previously described [49, 50].

We developed logistic regression models to examine the independent associations of having had “any complication” (yes/no) and having received ANC (yes/no), and of their interaction, with hospital delivery, separately for stillbirths and neonatal deaths. Models were developed to examine different aspects of ANC, including four or more visits (ANC4+); “quality ANC” (Q-ANC) consisting of six recommended interventions (blood pressure measurement; urine and blood sample tests; and counseling on proper nutrition, pregnancy danger signs, and where to go for any complication) over the course of all visits [51, 52]; counseling on danger signs and where to go, without necessarily receiving Q-ANC (DS-ANC); and receiving only one or more of the other four interventions (O-ANC). Potential confounders included in all models were residence (urban/rural), mother’s formal education level (none/some primary/some secondary or higher), and travel time to the nearest health facility in an emergency (less than 30 min/30 minutes or more).

Lastly, we conducted analyses to assess whether a delay in conducting C-section might have contributed to antepartum or intrapartum stillbirth or neonatal death. For each of these outcomes we examined labor duration and, by logistic regression, the association of hours before and after reaching the birth attendant with cesarean vs. vaginal delivery, adjusted for the presence of “any complication” (yes/no). Poisson regression was used to estimate the relative difference (RD) in labor duration of cesarean and vaginal deliveries. We used Poisson regression as an alternative to linear regression, assuming that long delivery times were more variable than short ones.

## Results

Of 851 TDHS-identified stillbirths and under-5-years deaths, 783 (92.0%) had a VASA interview completed. Additional file 1: Table S1 shows the VASA and TDHS classifications of all 783 deaths. The current analysis is of the 204 stillbirths and 228 neonatal deaths identified by the VASA. Most VASA respondents for stillbirths (90.3%) and neonatal deaths (92.5%) were the deceased’s mother. The recall period between the dates of death and interview varied from 1 to 7 years (median 4, IQR 3, 5) both for stillbirths and neonates.

### Demographic characteristics

Pregnancy duration of stillbirths and neonatal deaths was similar (Table 1). Nearly half of the neonates died within 24 h of delivery, and nine-tenths in the first week. There was a male predominance both of stillbirths and neonatal deaths. Mothers’ mean age and years of schooling for stillbirths and neonatal deaths were similar, and residence for both was mainly rural.

**Table 1:** Demographic characteristics of 204 stillbirths and 228 neonatal deaths, Tanzania, 08/2011 to 02/2016

Characteristic	Stillbirths	Neonatal deaths
Pregnancy duration (months)		
6	NA^a^	7 (3.0%)
7	26 (12.7%)	33 (14.6%)
8	41 (19.9%)	33 (14.4%)
9	131 (64.0%)	143 (62.9%)
10	7 (3.4%)	12 (5.1%)
Age (days)		
0	NA	104 (45.5%)
1–6	NA	99 (43.3%)
7–13	NA	14 (6.0%)
14–27	NA	12 (5.1%)
Sex		
Male	108 (53.1%)	129 (56.4%)
Female	93 (45.4%)	99 (43.6%)
Missing/Don’t know	3 (1.5%)	0 (0.0%)
Mother’s age at child’s birth (years)		
< 20	43 (21.2%)	51 (22.2%)
20–29	85 (41.4%)	110 (48.2%)
>=30	75 (36.8%)	63 (27.8%)
Missing/Don’t know	1 (0.6%)	4 (1.8%)
Mean (SE) age	26.9 (0.664)	26.1 (0.931)
Mother’s education		
None	32 (15.9%)	30 (13.0%)
Primary (some, grades 1–7)	145 (71.0%)	164 (72.1%)
Secondary+ (some, grades 8+)	27 (13.1%)	34 (14.9%)
Mean (SE) years of school	5.7 (0.231)	6.3 (0.274)
Residence		
Rural	154 (75.6%)	148 (64.9%)

^a^ *NA *Not applicable

### Antepartum and intrapartum stillbirth

Sufficient information to classify fetal deaths as antepartum or intrapartum, based solely on mother’s reports of fetal movement, was available for 185/204 (90.7%) stillbirths (Table 2). The distribution of these 185 by pregnancy duration was similar to that for all 204 stillbirths (full-term: 67.4%). However, more intrapartum than antepartum stillbirths were products of full-term pregnancies; and intrapartum stillbirths had significantly longer pregnancy duration.

**Table 2:** Pregnancy duration of 97 antepartum and 91 intrapartum stillbirths, Tanzania, 08/2011 to 02/2016

Pregnancy duration (months)	Total	Intrapartum	Antepartum	X^2^, *p* ^a^	
7	24 (13.1%)	15 (15.9%)	9 (10.1%)		17.03, < 0.001
8	38 (20.3%)	9 (9.0%)	29 (32.8%)		
9	120 (64.6%)	70 (71.6%)	50 (56.9%)	4.16, 0.042	
10	4 (2.0%)	3 (3.5%)	0 (0.2%)		
Total	185 (100%)	97 (52.5%)	88 (47.5%)		

^a^Rao-Scott chi-square

Including mothers’ reports of maceration in the VA definitions of intrapartum and antepartum stillbirth resulted in apparent over diagnosis of intrapartum stillbirth, with excessively long reported duration of no fetal movement before delivery without maceration (Additional file 1: Tables S2 and S3).

### Maternal complications

Several pregnancy complications trended in the expected direction of being positively associated with antepartum stillbirth, but only maternal infection and preeclampsia/eclampsia approached statistical significance. While some L/D complications were positively associated with intrapartum stillbirth, the associations were weak (Additional file 1: Table S4).

Table 3 shows the association of maternal complications with three major causes of early NNM. Antepartum hemorrhage (APH), maternal anemia, and premature rupture of membranes (PROM) were significantly positively associated with early NNM due to preterm delivery, intrapartum-related events, and serious infection, respectively.

**Table 3:** Association of maternal complications with three main causes of 195 early (days 0–6) neonatal deaths, Tanzania, 08/2011 to 02/2016

Early neonatal cause of death Maternal complications^a^	COD^b^ of interest n (%)	Other CODs n (%)	X^2^	p
**Preterm delivery**	***N* = 28**	***N*** **= 167 **		
Antepartum hemorrhage	5 (18.8)	11 (6.6)	4.98	0.026
Any of four complications	9 (32.1)	31 (18.9)	1.84	0.175
**Intrapartum-related events**	***N*** **= 57 **	***N*** **= 138 **		
Maternal anemia	8 (13.5)	6 (4.1)	4.85	0.028
Antepartum or intrapartum hemorrhage	16 (28.7)	34 (24.8)	0.29	0.591
Prolonged labor	22 (37.6)	41 (29.7)	0.75	0.388
Malpresentation	7 (12.7)	14 (10.6)	0.12	0.732
Cord complication	8 (13.8)	10 (7.4)	1.21	0.272
Any of eight complications	41 (70.7)	84 (61.3)	0.74	0.391
**Serious infection**	***N*** **= 60 **	***N*** **= 135 **		
Premature rupture of membranes	5 (8.1)	4 (2.7)	4.20	0.040
Prolonged labor	21 (34.8)	41 (30.8)	0.23	0.631
Any of three complications	24 (39.3)	49 (36.6)	0.10	0.753

^a^Only complications with five or more deaths for a COD of interest are displayed in the table. Other complications included in ‘any of (four/eight/three) complications’ are: for preterm delivery: maternal anemia, maternal infection, and premature rupture of membranes; for intrapartum-related events (birth asphyxia and birth injury): maternal infection, preeclampsia/eclampsia, and maternal diabetes; for serious infection (sepsis, pneumonia, or meningitis): maternal infection

^b^COD=cause of death (the causes of interest are preterm delivery, intrapartum-related events, and serious infection

Additional file 1: Tables S5 and S6 show that APH was also significantly associated with preterm delivery among all 228 neonatal deaths and among the 129 early onset (age 0–1 day) deaths.

### Maternal care: antenatal care

While 93% and 96% of women with a stillbirth and neonatal death, respectively, made at least one ANC visit, only 55% and 69% achieved ANC4 + and just 19% and 35% received Q-ANC. As seen in Table 4, in general, mothers who delivered in urban areas and in hospitals had relatively higher coverage.

**Table 4:** Coverage of key interventions during the antenatal period, by residency and delivery place

Intervention	Stillbirths (*N* = 204)				Neonatal deaths (*N* = 228)			
	Urban (*n* = 50)	Rural (*n* = 154)	Hospital delivery (*n* = 91)	Other delivery places (*n* = 113)	Urban (80)	Rural (148)	Hospital delivery (105)	Other delivery place (123)
At least one ANC^a^ visit	97.7%	91.6%	97.9%	89.2%	91.9%	97.6%	98.8%	92.9%
At least 4 ANC visits	73.3%	49.4%	78.0%	37.1%	75.9%	64.7%	79.8%	59.2%
Quality ANC^b^	40.3%	12.2%	30.8%	10.0%	47.2%	29.5%	48.4%	24.7%

^a^ *ANC *Antenatal care

^b^Quality ANC includes blood pressure checked, urine and blood tested, counseled about nutrition, and counseled about pregnancy danger signs and where to go in case of any danger sign

Mothers of stillbirths and neonatal deaths who achieved ANC4 + were, respectively, nearly three times (OR = 2.72, 95% CI 1.24, 6.00, *p* = 0.014) and twice (OR = 2.18, 95% CI 0.93, 5.06, *p* = 0.074) as likely to receive Q-ANC as their counterparts with less than four visits.

### Maternal care: careseeking for complications

Somewhat more mothers of stillbirths (61/204 [29.9%]) than neonatal deaths (47/228 [20.8%]) had a pregnancy complication. However, women with a neonatal death sought health care for these complications significantly more often (43/47 [91.6%] vs. 36/61 [58.4%], X^2^ = 21.34, *p* < 0.001), with this difference being driven by careseeking for APH (19/19 [100%] vs. 18/27 [69.0%], X^2^ = 26.68, *p* < 0.001). Nearly half the mothers of stillbirths (101/204 [49.4%]) and neonatal deaths (100/228 [43.8%]) had a L/D complication that began before reaching a delivery facility, but with no differences in careseeking.

### Maternal care: delivery place and mode

When adjusted by logistic regression, urban residence was strongly predictive of hospital delivery both for neonates and stillbirths; while achieving ANC4 + increased hospital delivery for stillbirths but not neonates, and having any complication did not increase hospital delivery for neonates or stillbirths (Additional file 1: Tables S7 and S8).

**Fig. 1:**
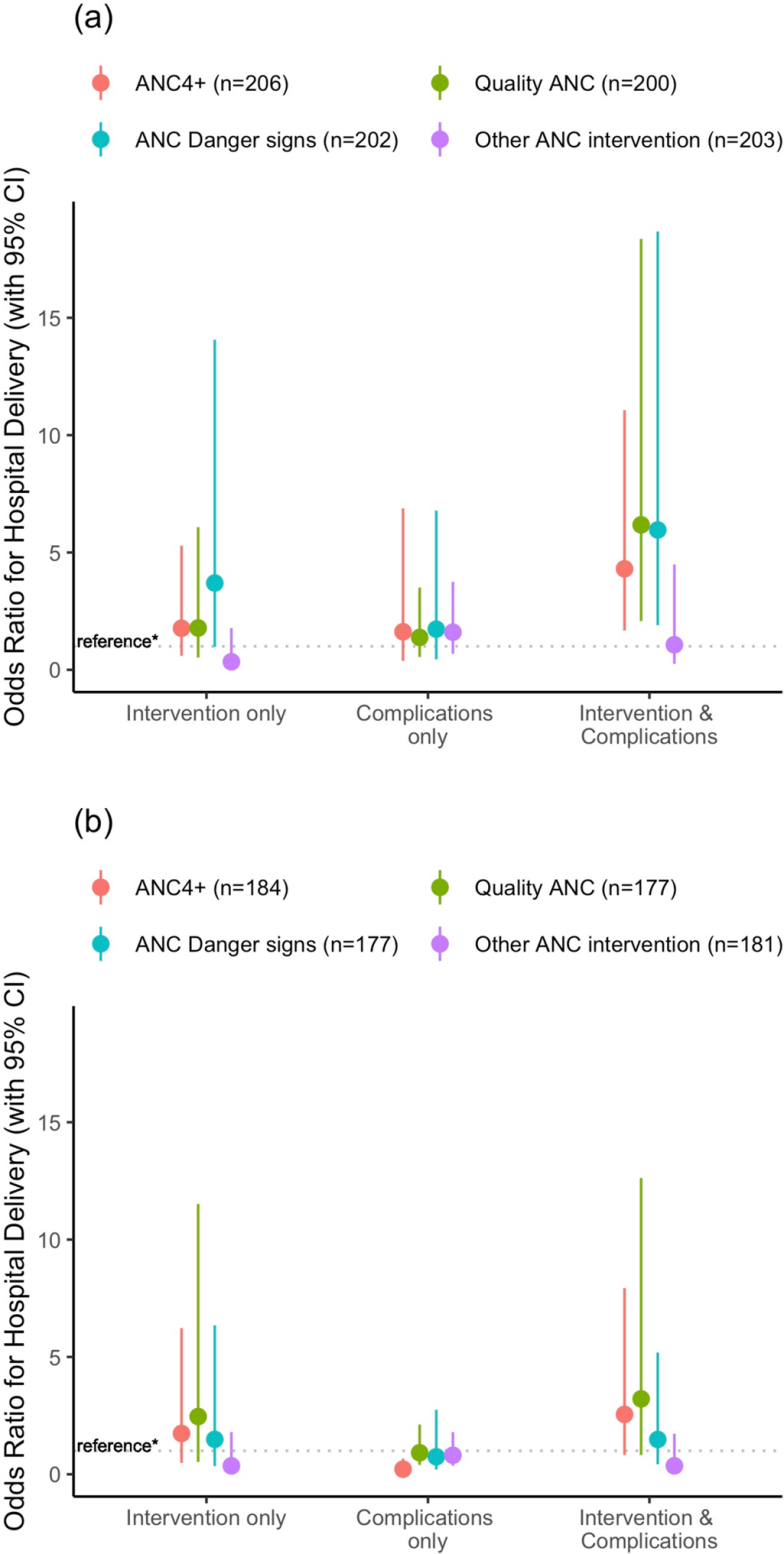
Interaction of maternal complications and antenatal care on hospital delivery. Logistic regression models adjusted for urban/rural residence, mother’s education, and travel time to the nearest health facility in an emergency: (**a**) neonatal death, (**b**) stillbirth. ANC: Antenatal care; ANC4+: Four or more ANC visits; Quality ANC: ANC with six interventions, including counseling on pregnancy danger signs; ANC Danger signs: ANC with counseling on pregnancy danger signs, without necessarily receiving Quality ANC; Other ANC intervention: ANC with one or more of four interventions (blood test, blood pressure taken, urine test, counseling on nutrition), without counseling on pregnancy danger signs; *reference: No intervention or complications

 Nevertheless, having any complication and achieving ANC4 + interacted to more than quadruple hospital delivery of neonates compared to women without a complication and fewer than four ANC visits (Fig. 1a, Additional file 1: Table S7). Women with any complication who received Q-ANC or DS-ANC were even more likely to deliver in hospital, while having any complication and receiving O-ANC had no effect on hospital delivery. This differed for stillbirths, for whom both having any complication and receiving any of the three ANC types, vs. not having a complication nor any of the ANC types, did not increase hospital delivery; while having any complication without ANC4 + decreased hospital delivery by four-fifths (Fig. 1b, Additional file 1: Table S8).

Not depicted in Fig. 1b is that women with a stillbirth who had any complication and achieved ANC4 + were highly more likely to deliver in hospital than women with a complication who made fewer than four visits (Additional file 1: Table S8). Women with a neonatal death who had any complication similarly had increased hospital delivery if they received Q-ANC or DS-ANC, compared to women with any complication who did not receive these ANC types (Additional file 1: Table S7).

Fifty-eight (47%) of the 123 women with a stillbirth and any complication said they had a careseeking constraint, the most common being the cost for transportation or health care (20%), thinking they did not need care (17%), lack of transportation (12%), and distance (10%). Twelve (20%) of these 58 delivered at hospital, compared to 37 (57%) of the 66 women without a constraint (OR = 0.19, 95% CI 0.08, 0.44, *p* < 0.001). Sixty-one other women with a stillbirth had one or more symptoms such as blurred vision and fever that did not meet the criteria for an obstetric complication. Three (44%) of the eight such women with a careseeking constraint delivered at hospital, vs. 25 (48%) of the 53 without a constraint (OR = 0.85, 95% CI 0.12, 5.81, *p* = 0.864).

16% (32) of stillbirths and 10% (23) of neonatal deaths were delivered by C-section, all at hospital except one neonate. Among hospital deliveries, labor duration of neonates delivered by C-section (median 9.0 h, IQR 4.5, 19.5) and vaginally (median 9.0, IQR 3.0, 16.0) was similar (RD 1.34, 95% CI 0.77, 2.34, *p* = 0.298); while labor duration of intrapartum stillbirths delivered by C-section (median 16.5, IQR 5.5, 42.5) was prolonged vs. that of vaginal deliveries (median 10.0, IQR 5.0, 18.0) (RD 2.51, 95% CI 1.30, 4.86, *p* = 0.007).

The time after reaching the birth attendant contributed half of the total labor duration of women with an intrapartum stillbirth (median 0.50, IQR 0.11, 0.84). After adjusting for hours of labor before reaching the birth attendant and presence of any complication, women with an intrapartum stillbirth were 6.5% (aOR = 1.065, 95% CI 1.002, 1.132, *p* = 0.044) more likely to have a C-section for every additional hour before delivery after reaching the attendant. Adjusted for the labor duration phases, having any complication conferred no risk (aOR = 0.963, 95% CI 0.049, 18.778, *p* = 0.980).

## Discussion

Stillbirth and neonatal death represent a major public health problem in many low- and lower middle-income countries, with economic, social, and health implications for families and society [53]. Continued high mortality levels in Tanzania have been attributed to insufficient coverage and inequitable provision of BEmONC and CEmONC services in rural areas and by SES [21, 22]. The current paper, based on analyses of the 2017-18 Tanzania VASA study, provides additional evidence that the country can apply in its effort to improve maternal and newborn health programming.

For the first time in Tanzania, we directly estimated the national proportion of all stillbirths that are intrapartum. The level determined, 52.5%, closely agrees with a prior 51.1% [4] indirect estimate for SSA. We based our determination solely on mothers’ reports of fetal movement and found that including reports of maceration resulted in apparent over diagnosis of intrapartum stillbirth, with prolonged lack of fetal movement without maceration. “No maceration” has often been included as a VA criterion of intrapartum stillbirth [54]. However, pathology and VA studies have found poor agreement, respectively, between health providers’ assessment of maceration and time since fetal death [55, 56] and mothers’ reports of maceration and fetal movement [57–59]. Studies have also found high levels and variability of respondent uncertainty regarding the presence of maceration as compared to clarity and consistency of reports of fetal movement [59], as well as up to 4.5 times risk of (antepartum) stillbirth in women who report decreased fetal movement before labor onset [60, 61]. Other VA studies have similarly given preference to mothers’ reports of fetal movement in distinguishing antepartum from intrapartum stillbirth [62].

Significantly more intrapartum than antepartum stillbirths being full term strengthens the certainty of our definition, since many intrapartum stillbirths are expected of full-term fetuses dying from intrapartum-related events. The importance of this finding is the possibility of decreasing intrapartum stillbirths, which are of longer gestation and have a greater chance of survival through early detection of fetal distress, conduct of C-section, and newborn resuscitation [63]. Our finding for intrapartum stillbirths of median 16.5 h labor duration, with the period from reaching the birth attendant until delivery significantly associated with C-section, suggests that delay in conducting C-section contributed to the deaths. Inadequate availability of general anesthesia equipment has been identified as the main roadblock to timely conduct of C-section in Tanzania [64].

Positive associations identified between APH, maternal anemia, and PROM and, respectively, preterm delivery, intrapartum-related events, and serious neonatal infection can provide guidance in strengthening Tanzania’s 2016–2020 One Plan II and 2021/22-2025/26 One Plan III program updates of its 2008–2015 One Plan program. Utilizing this information for evidence-based quality improvement of service delivery through clinical mentorship and supportive supervision, especially in low performing regions, fits perfectly with the One Plan II and III’s implementation strategies and guiding principles [22, 65].

Although maternal infection is thought to be the cause of up to 40% of spontaneous preterm births without PROM [39] and subsequent neonatal morbidity and mortality, we did not find an association between maternal infection and early NNM due to preterm delivery. Vertically transmitted maternal infection can also be the cause of early onset neonatal sepsis [39, 66], but we did not find this association among 195 early neonatal deaths, nor among all 228 neonatal deaths or 129 early onset deaths (data not shown). This may be because intrauterine infection causing preterm birth and neonatal sepsis is often asymptomatic [39], and because other maternal genitourinary infections implicated in preterm birth, including bacterial vaginosis and asymptomatic bacteriuria, are not detected by our VA algorithms.

The gap in ANC4 + coverage of mothers of stillbirths (45%) and neonatal deaths (31%) represents an improvement over the 2010 TDHS’s 57% for all pregnant women [19]. This could be due to health sector reforms undertaken by Tanzania during the last decade to expand access to health services. However, continued concerns about quality [21, 67] and urban/rural disparities in access to delivery services [21] temper this conclusion. The positive association between ANC4 + and Q-ANC and the fact that only one-fifth to one-third of women received Q-ANC highlights the country’s need to further strengthen ANC quality, access and coverage.

Women with a neonatal death and any complication were no more likely to deliver in hospital than women without a complication unless they had achieved ANC4 + or received Q-ANC or DS-ANC. These findings were less clear for women with a stillbirth and any complication. Like women with a neonatal death and a complication, they were more likely to deliver in hospital if they achieved ANC4 + than if not. However, without ANC4 + they were only one-fifth as likely to deliver in hospital as women without a complication nor ANC4+, and also were not more likely to deliver in hospital if they received Q-ANC or DS-ANC. This differs from findings in Ghana [15], which suggested that Q-ANC decreased stillbirth by promoting health facility delivery. The tendency in our study for non-hospital delivery by women with a stillbirth and any complication might be explained by their higher reported level of careseeking constraints and lower level of careseeking in the face of a constraint.

Aligned with the Tanzanian Countdown Study [21] finding of a large urban/rural disparity in the proportion of births conducted in health facilities, the VASA study found that urban residence was the strongest predictor of hospital delivery both for neonatal deaths and stillbirths. The Countdown Study found inequity in coverage and quality of delivery services in rural areas to be a factor in Tanzania’s slow decline in PNM and NNM. However, both indicators remain higher in urban (PNM: 47/1,000; NNM: 63/1,000) than rural (PNM: 37/1,000; NNM: 47/1,000) Tanzania [20], with only inconclusive explanations why this is so [68, 69]. A prospective cohort study of pregnant women in rural Tanzania found more L/D complications among women who delivered at facility than at home and, when controlled for complications, PNM was higher among facility births [70]. The authors attributed their findings to the need for improved training in recognizing and managing complications and supplying facilities with essential drugs and equipment. It is reasonable to hypothesize that a similar situation pertains in urban areas, with more women with complications, encouraged by ANC4 + and Q-ANC, delivering in facilities ill-equipped to manage their complications. In such a scenario, high levels of facility delivery in urban areas might even contribute to their higher PNM and NNM levels. Further study of the quality of care provided in urban delivery facilities, beyond the scope of the VASA study, is needed to assess this hypothesis.

### Limitations

VASA study limitations have been discussed elsewhere [50]. Verbal autopsy diagnoses, while currently the most accurate possible at population level in low- and lower middle-income countries, are not as accurate as medical diagnoses with direct measurement. This could possibly result in some inaccuracy in our assessments of association between maternal complications and causes of neonatal death. Also, our interview-based measure of some Q-ANC components may overestimate true quality, since we were not able to determine if health care workers acted on abnormal findings, for example, of blood pressure or hematocrit. There could be recall bias due to the recall period of 1–7 years. Most respondents were the deceased’s mother, who may have provided socially desirable answers to sensitive questions. For example, this might have contributed to the higher reported level of careseeking constraints among women with a stillbirth and any complication who did not seek care. This concern is moderated by the finding that women with similar symptoms that did not qualify as a complication were as likely to deliver at hospital whether or not they reported careseeking problems.

Our separate analyses and somewhat different findings for stillbirths and neonatal deaths rest on the ability of VA to distinguish these birth outcomes [71, 72]. Asking about vital signs present at birth, the method used by our VASA study, is assumed to be superior to the usual survey methods of asking a full birth history or full pregnancy history [59, 71]. A comparison of the full birth history and full pregnancy history methods, in which the full pregnancy history asked about signs of life only for babies reported to be born dead found that the full pregnancy history identified more stillbirths but did not decrease misclassification between stillbirths and early neonatal deaths [73]. However, the VASA study asked about signs of life both for babies reported to be born alive and dead, and so might be expected to perform better in this regard.

The statistical power of some of our analyses was restricted by sample size. The positive associations of maternal complications with neonatal causes of death are based on few cases, yet nevertheless yielded significant findings. Additional file 1: Tables S7 and S8 show the n/N of women in each ANC category who delivered at hospital to enable the reader to consider the statistical power. Inclusion of a control group would have enabled assessment of differences in ANC coverage, the level of complications, and hospital delivery among mothers of cases (stillbirths or neonatal deaths) and controls (surviving neonates). However, the lack of a comparison group in VASA studies is common and not so necessary since they examine interventions with proven effectiveness against NNM [74] that should be accessible to all pregnant women and newborns.

## Conclusions

While our study demonstrated the ability of Q-ANC and ANC4 + to increase hospital delivery by women with complications, urban residence was the strongest predictor of hospital delivery, and the quality of delivery and neonatal care provided by facilities in all areas is clearly as important as coverage. The VASA study identified complications significantly associated with leading causes of NNM in Tanzania and demonstrated that intrapartum stillbirths were most often full term and likely contributed to by provider delay in conducting C-sections. This information can be used to help focus training of personnel and appropriate supplying and equipping of facilities. Pregnancy complications were highly prevalent among mothers of stillbirths and neonatal deaths in the VASA study and a small minority of women received Q-ANC. Increased coverage of ANC4 + and Q-ANC, especially of WHO’s focused ANC model [52] adopted by Tanzania in 2002, which includes detection, management and, when necessary, referral to specialty care of women with complications, could also contribute to decreasing perinatal and neonatal mortality.

Our analysis also suggests that, within the context of a VA- or survey-based evaluation, maternal assessment of fetal movement, without consideration of maceration, is the more reliable means of distinguishing intrapartum from antepartum stillbirth.

### Supplementary Information

**Additional file 1:** **Table S1. **2015/16 TDHS and 2017/18 Tanzania VASA study classification of status at birth of 783 stillbirths, neonatal and child deaths from 08/2011 to 02/2016.** Table S2. **Intrapartum and antepartum stillbirths defined with and without mothers reports of fetal maceration in relation to fetal movement less than 8 hours before delivery or before the onset of labor, Tanzania, 08/2011 to 02/2016.** Table S3.** Intrapartum and antepartum stillbirths defined with and without mothers reports of fetal maceration in relation to fetal movement less than 12 hours before delivery or before the onset of labor, Tanzania, 08/2011 to 02/2016. **Table S4.** Association of maternal complications with 185 intrapartum and antepartum stillbirths, Tanzania, 08/2011 to 02/2016. **Table S5.** Association of selected maternal complications with three main causes of 228 neonatal (days 0-27) deaths, Tanzania, 08/2011 to 02/2016. **Table S6.** Association of selected maternal complications with three main causes of 129 early-onset (days 0-1) neonatal deaths, Tanzania, 08/2011 to 02/2016. **Table S7.** Logistic regression model of the independent effects of four or more antenatal care visits and one or more maternal complications on hospital delivery of neonates that died; and models that include the same potential confounders, showing the effect of the interaction of different aspects of antenatal care and complications on hospital delivery. **Table S8.** Logistic regression model of the independent effects of four or more antenatal care visits and one or more maternal complications on hospital delivery of stillbirths; and models that include the same potential confounders, showing the effect of the interaction of different aspects of antenatal care and complications on hospital delivery.

## Data Availability

The participant-de-identified VASA dataset analyzed during the current study will be made available at an open-access URL upon publication. The anonymized VASA dataset analyzed for the current study and associated questionnaire and variables mapping guide can be found at the following Tanzania National Bureau of Statistics website: https://www.nbs.go.tz/index.php/en/#/Databases and at the following Johns Hopkins sharepoint site: 2017 Tanzania VASA.zip.

## Abbreviations

aOR: Adjusted odds ratio
ANC: Antenatal care
ANC4+: Four or more antenatal care visits
APH: Antepartum hemorrhage
BEmONC: Basic emergency obstetric and neonatal care
CEmONC: Comprehensive emergency obstetric and neonatal care
CI: Confidence interval
COD: Cause of death
DS-ANC: Antenatal care that includes counseling on pregnancy danger signs
IQR: Interquartile range
L/D: labor and delivery
NA: Not applicable
NNM: Neonatal mortality
O-ANC: Antenatal care that includes only one or more of four interventions (blood test, blood pressure taken, urine test, counseling on nutrition) other than counseling on pregnancy danger signs
OR: Odds ratio
PNM: Perinatal mortality
PROM: Premature rupture of membranes
RD: Relative difference
SES: Socioeconomic status
SSA: Sub-Saharan Africa
Q-ANC: Quality antenatal care
TDHS: Tanzania Demographic and Health Survey
U5M: Under-5 mortality
VASA: Verbal and social autopsy
