# Supplementary material for: Maternal interventions to decrease stillbirths and neonatal mortality in Tanzania: evidence from the 2017-18 cross-sectional Tanzania verbal and social autopsy study
Source: BMC Pregnancy Childbirth. 2023 Dec 11;23:849. doi: 10.1186/s12884-023-06099-y (PMC10714492; doi:10.1186/s12884-023-06099-y)
Supplement: Supplementary file 1 — Additional file 1: Table S1. 2015/16 TDHS and 2017/18 Tanzania VASA study classification of status at birth of 783 stillbirths, neonatal and child deaths from 08/2011 to 02/2016. Table S2. Intrapartum and antepartum stillbirths defined with and without mothers reports of fetal maceration in relation to fetal movement less than 8 hours before delivery or before the onset of labor, Tanzania, 08/2011 to 02/2016. Table S3. Intrapartum and antepartum stillbirths defined with and without mothers reports of fetal maceration in relation to fetal movement less than 12 hours before delivery or before the onset of labor, Tanzania, 08/2011 to 02/2016. Table S4. Association of maternal complications with 185 intrapartum and antepartum stillbirths, Tanzania, 08/2011 to 02/2016. Table S5. Association of selected maternal complications with three main causes of 228 neonatal (days 0-27) deaths, Tanzania, 08/2011 to 02/2016. Table S6. Association of selected maternal complications with three main causes of 129 early-onset (days 0-1) neonatal deaths, Tanzania, 08/2011 to 02/2016. Table S7. Logistic regression model of the independent effects of four or more antenatal care visits and one or more maternal complications on hospital delivery of neonates that died; and models that include the same potential confounders, showing the effect of the interaction of different aspects of antenatal care and complications on hospital delivery. Table S8. Logistic regression model of the independent effects of four or more antenatal care visits and one or more maternal complications on hospital delivery of stillbirths; and models that include the same potential confounders, showing the effect of the interaction of different aspects of antenatal care and complications on hospital delivery. [file 12884_2023_6099_MOESM1_ESM.docx]

**Additional file: Table S1: 2015/16 TDHS and 2017/18 Tanzania VASA study classification of status at birth of 783 stillbirths, neonatal and child deaths from 08/2011 to 02/2016**

**TDHS age-at-death VASA age-at-death**

**Stillbirth 0-27 days 28 days-59 months Total**

Stillbirth 184 13 0 197 (25.2%)

0-27 days 18 213 13 244 (31.2%)

28 days-59 months 2 2 338 251 (43.7%)

Total 204 (26.1%) 228 (29.1%) 351 (44.8%) 783 (100.0%)

**Additional file: Table S2: Intrapartum and antepartum stillbirths defined with and without mothers’ reports of fetal maceration in relation to fetal movement less than 8 hours before delivery or before the onset of labor, Tanzania, 08/2011 to 02/2016**

**Including maceration**

**in definition* Excluding maceration from definition**^β^

**Intrapartum Antepartum Total**

Intrapartum 97 33^±^ 130 (70.1%)

Antepartum 0 55 55 (29.9%)

Total 97 (52.5%) 88 (47.5%) 185 (100.0%)

VA definitions of intrapartum (‘fresh’) stillbirth: *Did not stop moving before labor began OR stopped moving <1 hour before labor began OR stopped moving <8 hours before delivery OR no maceration; ^β^Did not stop moving before labor began OR stopped moving <1 hour before labor began OR stopped moving <8 hours before delivery.

^±^These 33 stillbirths classified as intrapartum by way of no reported maceration had median reported time of no fetal movement before delivery of 49.0 hours (IQR 28.0, 176.0).

**Additional file: Table S3: Intrapartum and antepartum stillbirths defined with and without mothers’ reports of fetal maceration in relation to fetal movement less than 12 hours before delivery or before the onset of labor, Tanzania, 08/2011 to 02/2016**

**Including maceration**

**in definition* Excluding maceration from definition** ^β^

**Intrapartum Antepartum Total**

Intrapartum 102 29^±^ 131 (70.6%)

Antepartum 0 54 54 (29.4%)

Total 102 (55.1%) 83 (44.9%) 185 (100.0%)

VA definitions of intrapartum (‘fresh’) stillbirth: *Did not stop moving before labor began OR stopped moving <1 hour before labor began OR stopped moving <12 hours before delivery OR no maceration; ^β^Did not stop moving before labor began OR stopped moving <1 hour before labor began OR stopped moving <12 hours before delivery.

^±^These 29 stillbirths classified as intrapartum by way of no reported maceration had median reported time of no fetal movement before delivery of 52.0 hours (IQR 30.0, 176.0).

**Additional file: Table S4: Association of maternal complications with 185 intrapartum and antepartum stillbirths, Tanzania, 08/2011 to 02/2016**

**Intrapartum Antepartum**

**N=97 N=88**

**Maternal complication n (%) n (%) X^2^ p***

**Pregnancy complications**

Maternal infection 5 (5.5) 13 (14.3) 3.13 0.077

Antepartum hemorrhage 10 (10.7) 13 (15.3) 0.61 0.434

Premature rupture of membranes 8 (8.4) 5 (6.1) 0.18 0.671

Preeclampsia/eclampsia (antepartum) 2 (1.7) 6 (6.5) 3.82 0.051

Any pregnancy complication^§^ 27 (28.2) 29 (33.6) 0.48 0.490

**Labor/delivery complications**

Preeclampsia/eclampsia 2 (1.8) 6 (6.5) 3.57 0.059

Intrapartum hemorrhage 25 (26.2) 20 (22.4) 0.27 0.602

Prolonged labor 37 (38.5) 29 (33.2) 0.38 0.536

Malpresentation 14 (15.2) 20 (23.4) 1.20 0.274

Cord complication 10 (11.3) 4 (5.0) 1.17 0.280

Any labor/delivery complication^§^ 67 (68.5) 54 (61.0) 0.75 0.388

*Rao-Scott chi-square (all except with superscript^β^); ^β^Pearson chi-square; ^§^Only pregnancy complications with five or more antepartum stillbirths and labor/delivery complications with five or more intrapartum stillbirths are displayed in the table. Other complications included in ‘any pregnancy complication’ are: maternal anemia, maternal diabetes and malaria; and in ‘any labor/delivery complication’ are maternal infection and preeclampsia/eclampsia.

**Additional file: Table S5: Association of selected maternal complications with three main causes of 228 neonatal (days 0-27) deaths, Tanzania, 08/2011 to 02/2016**

**Neonatal cause of death COD of interest Other COD**

**Maternal complication n (%) n (%) X^2^ p^€^**

**Preterm delivery N=32 N=196**

Antepartum hemorrhage 6 (17.5) 14 (6.9) 4.20 0.041

**IPRE N=60 N=168**

Maternal anemia 8 (13.3) 8 (4.7) 3.01 0.083

**Serious infection N=78 N=150**

Premature rupture of membranes 5 (6.5) 4 (2.6) 2.54 0.111

COD=cause of death (the causes of interest are preterm delivery, IPRE, and serious infection); ^€^Rao-Scott chi-square; IPRE=Intrapartum-related event (birth asphyxia or birth injury); Serious infection=sepsis, pneumonia or meningitis.

**Additional file: Table S6: Association of selected maternal complications with three main causes of 129 early-onset (days 0-1) neonatal deaths, Tanzania, 08/2011 to 02/2016**

**Early onset neonatal cause of death COD of interest Other COD**

**Maternal complication n (%) n (%) X^2^ p^€^**

**Preterm delivery N=21 N=108**

Antepartum hemorrhage 5 (25.5) 7 (6.1) 8.98 0.003

**IPRE N=43 N=86**

Maternal anemia 6 (14.3) 3 (3.6) 3.74 0.053

**Serious infection N=40 N=89**

Premature rupture of membranes 2 (4.2) 3 (3.1) 0.172 0.678

COD=cause of death (the causes of interest are preterm delivery, IPRE, and serious infection); ^€^Rao-Scott chi-square; IPRE=Intrapartum-related event (birth asphyxia or birth injury); Serious infection=sepsis, pneumonia or meningitis.

**Additional file: Table S7: Logistic regression model of the independent effects of four or more antenatal care visits and one or more maternal complications on hospital delivery of neonates that died; and models that include the same potential confounders, showing the effect of the interaction of different aspects of antenatal care and complications on hospital delivery**

| **Logistic regression model** | **Comp0** | **ANC0** | **Comp1** | **ANC1** | **p-value** | **aOR** | **low** | **high** |
| --- | --- | --- | --- | --- | --- | --- | --- | --- |
| **ANC4+ without interaction terms (n/N=99/206)*** |  |  |  |  |  |  |  |  |
| Pregnancy or L/D complication | NA | NA | NA | NA | 0.07 | 2.15 | 0.94 | 4.92 |
| ANC4+ | NA | NA | NA | NA | 0.10 | 2.26 | 0.86 | 5.95 |
| Mother primary vs. 0 school | NA | NA | NA | NA | 0.01 | 0.26 | 0.08 | 0.83 |
| Mother secondary vs. 0 school | NA | NA | NA | NA |  | 1.08 | 0.19 | 6.10 |
| Emergency travel time <30 vs ≥30 minutes | NA | NA | NA | NA | 0.12 | 2.35 | 0.81 | 6.79 |
| Urban/rural residence | NA | NA | NA | NA | <0.0001 | 5.85 | 2.48 | 13.79 |
| **ANC4+/complications interactions (n/N=99/206)** |  |  |  |  |  |  |  |  |
| ANC4+ | 0 | 0 | 0 | 1 | 0.31 | 1.77 | 0.59 | 5.29 |
| ANC4+ | 0 | 0 | 1 | 0 | 0.51 | 1.62 | 0.38 | 6.88 |
| ANC4+ | 0 | 0 | 1 | 1 | 0.00 | 4.30 | 1.67 | 11.06 |
| ANC4+ | 1 | 0 | 1 | 1 | 0.18 | 2.66 | 0.63 | 11.21 |
| **Q-ANC/complications interactions (n/N=97/200)** |  |  |  |  |  |  |  |  |
| Quality ANC | 0 | 0 | 0 | 1 | 0.36 | 1.78 | 0.52 | 6.08 |
| Quality ANC | 0 | 0 | 1 | 0 | 0.50 | 1.38 | 0.54 | 3.51 |
| Quality ANC | 0 | 0 | 1 | 1 | 0.00 | 6.18 | 2.08 | 18.36 |
| Quality ANC | 1 | 0 | 1 | 1 | 0.00 | 4.48 | 1.64 | 12.28 |
|  |  |  |  |  |  |  |  |  |
| **DS-ANC/complications interactions (n/N=98/202)** |  |  |  |  |  |  |  |  |
| ANC Danger signs | 0 | 0 | 0 | 1 | 0.06 | 3.70 | 0.97 | 14.07 |
| ANC Danger signs | 0 | 0 | 1 | 0 | 0.43 | 1.73 | 0.44 | 6.79 |
| ANC Danger signs | 0 | 0 | 1 | 1 | 0.00 | 5.96 | 1.90 | 18.68 |
| ANC Danger signs | 1 | 0 | 1 | 1 | 0.03 | 3.44 | 1.13 | 9.38 |
|  |  |  |  |  |  |  |  |  |
| **O-ANC/complications interactions (n/N=99/203)** |  |  |  |  |  |  |  |  |
| Other ANC intervention | 0 | 0 | 0 | 1 | 0.20 | 0.34 | 0.07 | 1.77 |
| Other ANC intervention | 0 | 0 | 1 | 0 | 0.28 | 1.60 | 0.69 | 3.75 |
| Other ANC intervention | 0 | 0 | 1 | 1 | 0.93 | 1.07 | 0.25 | 4.49 |
| Other ANC intervention | 1 | 0 | 1 | 1 | 0.57 | 0.67 | 0.16 | 2.71 |

*n/N=hospital delivery/all neonatal deaths; Comp0=had no maternal complication; Comp1=had one or more complications; ANC0=did not receive antenatal care of the listed type; ANC1=received antenatal of the listed type; aOR=adjusted odds ratio; low=lower limit of 95% confidence interval; high=upper limit of 95% confidence interval; ANC4+=four or more ANC visits; L/D=labor or delivery; Quality ANC=received six recommended ANC interventions (blood pressure measurement; urine and blood sample tests; and counseling on proper nutrition, pregnancy danger signs, and where to go for any complication) over the course of all ANC visits; ANC Danger signs=received counseling on danger signs and where to go, without receiving quality ANC; Other ANC intervention=received only one or more of the other four interventions.

**Additional file: Table S8: Logistic regression model of the independent effects of four or more antenatal care visits and one or more maternal complications on hospital delivery of stillbirths; and models that include the same potential confounders, showing the effect of the interaction of different aspects of antenatal care and complications on hospital delivery**

| **Logistic regression model** | **Comp0** | **ANC0** | **Comp1** | **ANC1** | **p-value** | **aOR** | **low** | **high** |
| --- | --- | --- | --- | --- | --- | --- | --- | --- |
| **ANC4+ without interaction terms (n/N=83/184)*** |  |  |  |  |  |  |  |  |
| Pregnancy or L/D complication | NA | NA | NA | NA | 0.38 | 0.69 | 0.31 | 1.56 |
| ANC4+ | NA | NA | NA | NA | 0.00 | 5.39 | 2.17 | 13.40 |
| Mother primary v. 0 school | NA | NA | NA | NA | 0.51 | 0.63 | 0.25 | 1.61 |
| Mother secondary v. 0 school | NA | NA | NA | NA |  | 0.97 | 0.26 | 3.56 |
| Emergency travel time <30 vs ≥30 minutes | NA | NA | NA | NA | 0.52 | 0.73 | 0.27 | 1.92 |
| Urban/rural residence | NA | NA | NA | NA | 0.00 | 7.17 | 2.29 | 22.47 |
| **ANC4+/complications interactions (n/N=83/184)** |  |  |  |  |  |  |  |  |
| ANC4+ | 0 | 0 | 0 | 1 | 0.40 | 1.74 | 0.49 | 6.23 |
| ANC4+ | 0 | 0 | 1 | 0 | 0.01 | 0.22 | 0.07 | 0.67 |
| ANC4+ | 0 | 0 | 1 | 1 | 0.11 | 2.55 | 0.82 | 7.93 |
| ANC4+ | 1 | 0 | 1 | 1 | <0.0001 | 11.75 | 3.63 | 38.04 |
| **Q-ANC/complications interactions (n/N=79/177)** |  |  |  |  |  |  |  |  |
| Quality ANC | 0 | 0 | 0 | 1 | 0.25 | 2.46 | 0.53 | 11.51 |
| Quality ANC | 0 | 0 | 1 | 0 | 0.84 | 0.92 | 0.40 | 2.12 |
| Quality ANC | 0 | 0 | 1 | 1 | 0.09 | 3.21 | 0.82 | 12.63 |
| Quality ANC | 1 | 0 | 1 | 1 | 0.06 | 3.51 | 0.96 | 12.83 |
|  |  |  |  |  |  |  |  |  |
| **DS-ANC/complications interactions (n/N=79/177)** |  |  |  |  |  |  |  |  |
| ANC Danger signs | 0 | 0 | 0 | 1 | 0.59 | 1.49 | 0.35 | 6.35 |
| ANC Danger signs | 0 | 0 | 1 | 0 | 0.66 | 0.75 | 0.20 | 2.75 |
| ANC Danger signs | 0 | 0 | 1 | 1 | 0.54 | 1.48 | 0.42 | 5.19 |
| ANC Danger signs | 1 | 0 | 1 | 1 | 0.17 | 1.99 | 0.74 | 5.34 |
|  |  |  |  |  |  |  |  |  |
| **O-ANC/complications interactions (n/N=82/181)** |  |  |  |  |  |  |  |  |
| Other ANC intervention | 0 | 0 | 0 | 1 | 0.21 | 0.36 | 0.07 | 1.80 |
| Other ANC intervention | 0 | 0 | 1 | 0 | 0.60 | 0.81 | 0.36 | 1.80 |
| Other ANC intervention | 0 | 0 | 1 | 1 | 0.20 | 0.36 | 0.08 | 1.73 |
| Other ANC intervention | 1 | 0 | 1 | 1 | 0.28 | 0.45 | 0.10 | 1.91 |

*n/N=hospital delivery/all stillbirths; Comp0=had no maternal complication; Comp1=had one or more complications; ANC0=did not receive antenatal care of the listed type; ANC1=received antenatal of the listed type; aOR=adjusted odds ratio; low=lower limit of 95% confidence interval; high=upper limit of 95% confidence interval; ANC4+=four or more ANC visits; L/D=labor or delivery; Quality ANC=received six recommended ANC interventions (blood pressure measurement; urine and blood sample tests; and counseling on proper nutrition, pregnancy danger signs, and where to go for any complication) over the course of all ANC visits; ANC Danger signs=received counseling on danger signs and where to go, without receiving quality ANC; Other ANC intervention=received only one or more of the other four interventions.
